# Phenotypic characterization and principal component analyses of indigenous chicken breeds in Indonesia

**DOI:** 10.14202/vetworld.2021.1665-1676

**Published:** 2021-06-28

**Authors:** Dyah Maharani, Fatmawati Mustofa, Aprilianna Putri Z. N. L. Sari, Akhmad Fathoni, Heru Sasongko, Dwi Nur Happy Hariyono

**Affiliations:** 1 Department of Animal Breeding and Reproduction, Faculty of Animal Science, Universitas Gadjah Mada, Yogyakarta 55281, Indonesia; 2 Department of Animal Production, Faculty of Animal Science, Universitas Gadjah Mada, Yogyakarta 55281, Indonesia; 3 Department of Animal Science, Faculty of Agriculture, Universitas Khairun, Ternate 97719, Indonesia

**Keywords:** indigenous chickens, phenotypic variation, qualitative traits, quantitative traits

## Abstract

**Background and Aim:**

Understanding the phenotypic characteristics of indigenous livestock breeds is essential for their utilization and conservation. This study aimed to characterize indigenous chicken breeds in Indonesia based on phenotypic traits.

**Materials and Methods:**

Data on eight qualitative and 12 quantitative traits were recorded for 250 chickens from six breeds: Black Kedu, Gaga, Merawang, Nunukan, Pelung, and Sentul. Data were analyzed using descriptive statistics and one-way analysis of variance to test the effect of breed on observed traits. Moreover, principal component analysis (PCA) was conducted separately for each chicken breed. Data on quantitative traits were subjected to Kaiser-Meyer-Olkin, which was computed to test the sampling adequacy and the pattern of correlation among the traits, and Bathlett’s tests were used to assess the validity of the factor analysis of each of the datasets and determine whether the partial correlations among traits were small.

**Results:**

We found considerable phenotypic variation in both qualitative and quantitative traits among indigenous chicken breeds. Multicolored plumage (96.40%), wild plumage (39.20%), gold feather flick (51.20%), yellow shank (36.80%), single comb (80.80%), red comb (94.80%), red earlobe (77.60%), and orange eyes (61.60%) were the most common features in the indigenous chickens. In addition, breed had a significant effect on all the quantitative traits that were analyzed (p<0.05). There were higher mean values for all quantitative traits for Pelung chickens than other chickens. In addition, the overall mean values for all quantitative traits in Merawang chicken were intermediate between Pelung chickens and Black Kedu, Gaga, and Nunukan chickens. The PCA showed two principal factors extracted that accounted for 77.80% and 78.38% of the total variance in the original variables for males and females, respectively.

**Conclusion:**

In general, body weight and body measurements, except wattle length, were loaded in PC1 as the primary factors responsible for the variation. The phenotypic variation observed in indigenous chickens in Indonesia could provide valuable basic information for the design of selection and genetic improvement programs.

## Introduction

Indigenous domestic chickens, *Gallus gallus domesticus*, are an integral component of rural poultry production in many developing countries. In Indonesia, they represent 8.24% of the poultry population, with the current population being about 311.9 billion birds [1]. Despite their low contribution to total poultry production, indigenous chickens serve as an investment for rural households, in addition to their use as ornamentation and a source of protein. Indigenous chickens are widely distributed across different agro-ecologies and often reared under the traditional scavenging system by small-holder farmers with low input for healthcare, feeding, and housing. In addition, the indigenous chickens are known for their high tolerance of poultry diseases, good adaptive responses to local climatic conditions, and their ability to survive with feed that varies in quality and quantity, meaning that they require low input [2].

Due to unpredictable demands for poultry products and likely climatic variation in the future, as well as a necessity for the sustainable use of indigenous chickens, the evaluation and monitoring of the phenotypic characteristics of chickens are highly recommended. Phenotypic characterization is the first step required to inform the utilization and conservation of indigenous livestock breeds. It allows researchers to identify phenotypic variation present within and between breeds, which could be valuable for improvement and selection programs for particular economic traits [3]. Furthermore, efficient utilization of an indigenous breed depends primarily on accurate knowledge of its physical characteristics that distinguish it from other breeds or species. There are about 31 indigenous chicken breeds that have been recognized in Indonesia. Of these, 12 breeds are kept as ornamental chickens, 11 breeds are kept as layer chickens, four breeds are kept as broiler chickens, and the remaining breeds are non-descript [4]. These indigenous chickens play an important role in constituting genetic stock of animal genetic resources. However, information on the phenotypic characteristics of indigenous chickens in Indonesia is lacking, making genetic improvement, and selection programs for traits of economic importance a formidable task [5-7]. In developing countries, indigenous chickens are poorly characterized both phenotypically and genetically, and this has led to the loss of poultry genetic resources [8,9].

Therefore, there is a need to determine the phenotypic characteristics of indigenous chickens to sustainably utilize and conserve them. The characterization of breeds based on phenotypic performance is a useful approach because it is simple, easy, fast, and cost-effective. Therefore, this study was undertaken to phenotypically characterize indigenous chicken breeds in Indonesia.

## Materials and Methods

### Ethical approval

The experimental method was approved by Research Ethics Committee of the Faculty of Veterinary Medicine, Universitas Gadjah Mada (00033/EC-FKH/Eks./2021).

### Study period and areas

**Figure-1:**
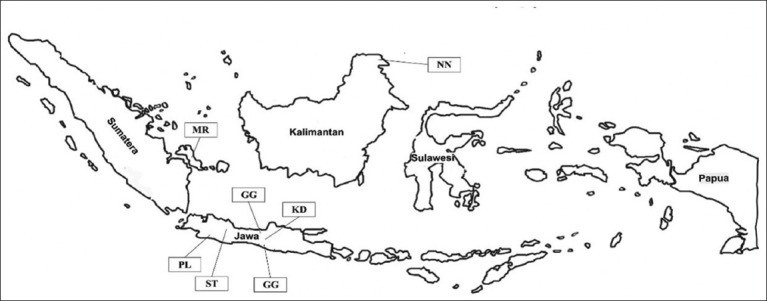
Location of analyzed six indigenous chicken populations in Indonesia (MR: Merawang; PL: Pelung; ST: Sentul; GG: Gaga; KD: Black Kedu; and NN: Nunukan) [Source: https://vemaps.com/indonesia/].

The study was conducted from July to December 2020. Six Indonesian indigenous chicken breeds, including Black Kedu, Gaga, Merawang, Nunukan, Pelung, and Sentul, were sampled from different regions. All the chicken breeds analyzed in this study are indigenous to Indonesia, but poorly investigated for their phenotypic characteristics. The details of sampling sites in this study are presented in Figure-1 and Supplementary Table-1.

### Experimental animals and their management

A total of 250 chickens (68 males and 182 females) were randomly sampled from the six Indonesian indigenous chicken breeds. Cockerels and hens were used for qualitative analysis for all breeds, except for Sentul chickens for which cockerels and pullets were used for qualitative analysis. Due to age differences, the Sentul chickens were excluded from the comparison of quantitative traits among the breeds. All the chickens analyzed were reared under controlled breeding systems at livestock breeding centers.

### Traits measured

All male and female chickens were individually assessed and scored for eight qualitative traits (plumage color, plumage pattern, feather flick, shank color, comb types, comb color, earlobe color, and eye color) and 12 quantitative traits (body weight, beak length, wattle length, breast width, breast circumference, wing length, breast length, femur length, tibia length, shank length, shank diameter, and third finger length), as per the method described by Cuesta [10] and Johari [11]. The qualitative traits for each chicken were visually appraised and scored. Body weight was measured using a digital scale, while the remaining quantitative traits were measured in centimeters using a measuring tape and Vernier caliper (0.01 mm). All measurements were taken by the same person to avoid individual variation during observation. All of the chicken breeds were included for the measurement of qualitative traits, whereas for quantitative traits, all breeds were included except Sentul chickens due to age differences between Sentul chickens and the remaining breeds.

### Statistical analysis

Data on qualitative traits were analyzed with descriptive statistics as percentages, while one-way analysis of variance was performed to analyze the effect of breeds on quantitative traits measured, followed by Duncan’s multiple range test to test significant differences between means (p<0.05).

The statistical model used was:

Yij=μ+G_i_+eij

where Yij is the individual body weight and body measurement, µ is the overall mean, Gi is the fixed effect of the breed, and eij is the random error.

Principal component analysis (PCA) represents linear combination of the available variables into factor or component. In this study, the PCA was performed separately for each chicken breed. PCA is a method for transforming the variables in a multivariate dataset X1, X2,…………Xp into new uncorrelated variables Y1, Y2,…………Yp, which account for decreasing proportions of the total variance of the original variables [12] defined as:

y_1_=a_11_x_1_+a_12_x_2_ +……………+a_1p_x_p_

y_2_=a_21_x_1_+a_22_x_2_+…………..+a_2p_x_p_

y_p_=a_p1_x_1_+a_p2_x_2_+…………..+a_pp_x_p_

with coefficients being chosen so that, y_1_, y_2_,……… y_p_ account for decreasing proportions of the total variance of the original variables x_1_, x_2_,…………x_p_. The PCA was performed using SPSS version 25.0 (SPSS Inc., Chicago, IL, USA) [13].

Data on quantitative traits were subjected to Kaiser-Meyer-Olkin (KMO) and Bathlett’s tests to assess the validity of the factor analysis of each of the datasets and to test whether the partial correlations among the traits were small. A KMO value of 0.50 and above was considered adequate [14].

## Results

### Variation in qualitative traits

**Figure-2:**
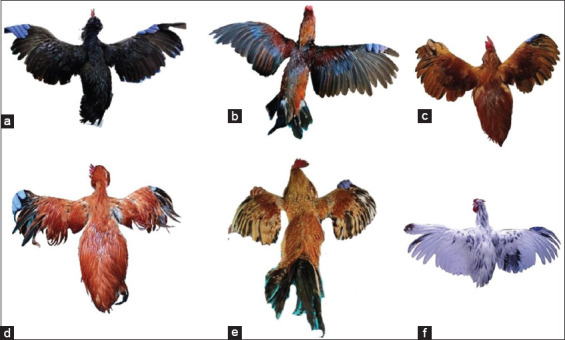
The plumage color variation of male indigenous chicken breeds in Indonesia. a. Black Kedu chicken, b. Gaga chicken, c. Merawang chicken, d. Nunukan chicken, e. Pelung chicken, f. Sentul Chicken.

**Figure-3:**
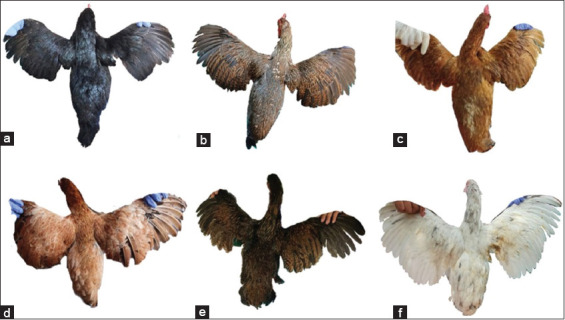
The plumage color variation of female indigenous chicken breeds in Indonesia. a. Black Kedu chicken, b. Gaga chicken, c. Merawang chicken, d. Nunukan chicken, e. Pelung chicken, f. Sentul Chicken.

Descriptive statistics for the qualitative traits of the six indigenous chicken breeds in Indonesia are presented in Table-1, and Figures-2 and 3 show plumage color variation of male and female indigenous chicken breeds, respectively. Two variants in plumage color, white, and multicolor, were observed across the chicken breeds. The majority of male (97.06%) and female (96.15%) chickens had multicolor plumage, while only 2.94% of males and 3.85% of females had white plumage (Table-1). Wild was the most common plumage pattern in the sampled chickens, being found in 39.20% of chickens on average, followed by Columbian (34.80%), black (23.20%), and white (2.80%). Two variants of feather flick (gold and silver) were observed. In male chickens, gold was the most prevalent, but it was least prevalent in female chickens. The most common shank color was yellow (35.29% in males and 37.36% in females), followed by gray-green, black, white, green, black-white, and black-yellow green.

**Table-1:** Variation of qualitative traits in Indonesian indigenous chickens.

Trait	Black Kedu		Gaga		Merawang		Pelung		Sentul		Overall		Overall (n=250)		
						
	Male (n=10)	Female (n=32)	Male (n=18)	Female (n=30)	Male (n=10)	Female (n=30)	Male (n=10)	Female (n=30)	Male (n=10)	Female (n=30)	Male (n=10)	Female (n=30)	Male (n=68)	Female (n=182)	
Plumage color															
White	0	0.00	5.56	10.00	0.00	0.00	0.00	0.00	10.00	0.00	0.00	13.33	2.94	3.85	3.60
Colored	100	100.00	94.44	90.00	100.00	100.00	100.00	100.00	90.00	100.00	100.00	86.67	97.06	96.15	96.40
Plumage pattern															
Black	100	100.00	0.00	13.33	0.00	0.00	0.00	0.00	0.00	40.00	0.00	0.00	14.71	26.37	23.20
White	0	0.00	0.00	6.67	0.00	0.00	0.00	0.00	10.00	0.00	0.00	13.33	1.47	3.30	2.80
Wild	0	0.00	100.00	80.00	0.00	0.00	0.00	0.00	30.00	56.67	100.00	86.67	45.59	36.81	39.20
Columbian	0	0.00	0.00	0.00	100.00	100.00	100.00	100.00	60.00	3.33	0.00	0.00	38.24	33.52	34.80
Feather flick															
Silver	100	100.00	16.67	46.67	0.00	0.00	0.00	0.00	30.00	83.33	50.00	100.00	30.88	55.49	48.80
Gold	0	0.00	83.33	53.33	100.00	100.00	100.00	100.00	70.00	16.67	50.00	0.00	69.12	44.51	51.20
Shank color															
White	0	0.00	0.00	10.00	0.00	0.00	0.00	3.33	30.00	6.67	50.00	20.00	11.76	6.59	8.00
Yellow	0	0.00	5.56	0.00	100.00	100.00	100.00	96.67	0.00	0.00	30.00	30.00	35.29	37.36	36.80
Grey-green	40	40.63	72.22	50.00	0.00	0.00	0.00	0.00	30.00	50.00	0.00	10.00	29.41	25.27	26.40
Green	0	9.38	22.22	36.67	0.00	0.00	0.00	0.00	0.00	0.00	0.00	0.00	5.88	7.69	7.20
Black	60	43.75	0.00	0.00	0.00	0.00	0.00	0.00	40.00	33.33	20.00	36.67	17.65	19.23	18.80
Black white	0	6.25	0.00	3.33	0.00	0.00	0.00	0.00	0.00	6.67	0.00	3.33	0.00	3.30	2.40
Black yellow green	0	0.00	0.00	0.00	0.00	0.00	0.00	0.00	0.00	3.33	0.00	0.00	0.00	0.55	0.40
Comb type															
Pea	0	0.00	0.00	0.00	0.00	0.00	0.00	0.00	0.00	0.00	30.00	83.33	4.41	13.74	11.20
Walnut	0	0.00	0.00	0.00	0.00	0.00	0.00	0.00	0.00	0.00	20.00	0.00	2.94	0.00	0.80
Single	100	100.00	72.22	63.33	100.00	100.00	100.00	100.00	100.00	100.00	30.00	16.67	82.35	80.22	80.80
Rose	0	0.00	27.78	36.67	0.00	0.00	0.00	0.00	0.00	0.00	20.00	0.00	10.29	6.04	7.20
Comb color															
Red	100	81.25	100.00	93.33	100.00	100.00	100.00	100.00	100.00	83.33	100.00	100.00	100.00	92.86	94.80
Red-black	0	18.75	0.00	6.67	0.00	0.00	0.00	0.00	0.00	16.67	0.00	0.00	0.00	7.14	5.20
Black	0	0.00	0.00	0.00	0.00	0.00	0.00	0.00	0.00	0.00	0.00	0.00	0.00	0.00	0.00
Earlobe color															
Red	80	53.13	66.67	76.67	100.00	86.67	50.00	80.00	50.00	80.00	100.00	100.00	73.53	79.12	77.60
Red-white	20	15.63	33.33	20.00	0.00	13.33	50.00	20.00	50.00	10.00	0.00	0.00	26.47	13.19	16.80
Red-black	0	31.25	0.00	3.33	0.00	0.00	0.00	0.00	0.00	10.00	0.00	0.00	0.00	7.69	5.60
Eye color															
Orange	0	34.38	94.44	86.67	100.00	80.00	80.00	63.33	60.00	36.67	90.00	43.33	73.53	57.14	61.60
Brown	100	62.50	5.56	13.33	0.00	0.00	0.00	16.67	30.00	63.33	10.00	53.33	22.06	35.16	31.60
Red	0	0.00	0.00	0.00	0.00	0.00	0.00	0.00	10.00	0.00	0.00	0.00	1.47	0.00	0.40
Pearl	0	3.13	0.00	0.00	0.00	20.00	20.00	20.00	0.00	0.00	0.00	3.34	2.94	7.70	6.40

Four comb types were observed across the six breeds: Pea, walnut, single, and rose. The majority of chickens (above 80.00%) had a single comb across the breeds and in both males and females. Notably, 100% of Black Kedu, Merawang, Gaga, and Pelung chickens had a single comb. All males and 92.86% of female chickens had a red comb. Variations in this trait were only observed in Black Kedu, Gaga, and Pelung chickens. Most male (73.53%) and female (79.12%) chickens had red earlobes. Variations in eye color were observed across the chicken breeds. Orange was the most predominant eye color across the breeds, with 75.53% in males and 57.14% in females, followed by brown, red, and pearl.

### Variation in quantitative traits

Descriptive statistics for all quantitative traits of indigenous chickens in Indonesia are shown in Table-2. There was a significant (p<0.05) effect of breed on all quantitative traits. Of the breeds analyzed, Pelung chickens showed the highest mean values for all quantitative traits, for both males and females. The mean values for all quantitative traits in Merawang chickens, for both males and females, were intermediate between Pelung chickens and Black Kedu, Gaga, and Nunukan chickens. The overall means of body weight, beak length, wattle length, breast width, breast circumference, wing length, breast length, femur length, tibia length, shank length, shank diameter, and third finger length in male and female chickens are presented in Table-2.

**Table-2:** Mean, standard errors, and coefficient of variation of quantitative traits measured among Indonesian indigenous chickens.

Trait	Sex	Breed									Overall		

		Black Kedu		Gaga		Merawang		Nunukan	Pelung				
					
		Mean±SE	CV	Mean±SE	CV	Mean±SE	CV	Mean±SE	CV	Mean±SE	CV	Mean±SE	CV
Body weight (kg)	Male	1.96±0.05^c^	0.16	2.07±0.06^c^	0.26	2.76±0.07^b^	0.23	1.65±0.13^d^	0.42	3.55±0.16^a^	0.52	2.35±0.10	0.73
	Female	1.61±0.06^c^	0.02	1.31±0.04^d^	0.20	2.14±0.05^b^	0.29	1.26±0.05^d^	0.26	3.08±0.15^a^	0.83	1.87±0.06	0.80
Beak length (cm)	Male	1.84±0.08^c^	0.24	2.07±0.05^b^	0.22	2.37±0.06^a^	0.21	2.18±0.06^b^	0.20	2.52±0.06^a^	0.20	2.18±0.04	0.31
	Female	1.80±0.04^c^	0.21	1.99±0.04 ^b^	0.21	2.08±0.02^b^	0.12	1.99±0.03^b^	0.14	2.37±0.04^a^	0.23	2.04±0.02	0.26
Wattle length (cm)	Male	3.86±0.22^b^	0.71	4.74±0.20^a^	0.86	4.83±0.31^a^	0.98	3.06±0.25^b^	0.80	5.38±0.43^a^	1.37	4.42±0.16	1.20
	Female	1.67±0.07^a^	0.40	1.74±0.10^a^	0.53	1.94±0.10^a^	0.56	1.19±0.07^b^	0.38	1.80±0.11^a^	0.60	1.66±0.04	0.55
Breast width (cm)	Male	7.86±0.13^c^	0.42	7.16±0.11^d^	0.47	8.41±0.07^b^	0.23	6.56±0.27^e^	0.86	9.78±0.22^a^	0.70	7.84±0.16	1.20
	Female	6.57±0.72^c^	0.41	6.30±0.07^cd^	0.38	7.28±0.10^b^	0.56	6.10±0.11^d^	0.62	8.53±0.14^a^	0.79	6.95±0.08	1.04
Breast circumference (cm)	Male	25.89±0.37^d^	1.16	28.87±0.52^c^	2.21	31.87±0.57^b^	1.82	25.75±0.81^d^	2.55	35.23±0.52^a^	1.63	29.43±0.51	3.91
	Female	25.52±0.34^c^	1.91	25.65±0.27^c^	1.48	28.42±0.31^b^	1.91	24.92±0.46^c^	2.50	35.57±0.61^a^	3.35	27.98±0.37	4.56
Wings length (cm)	Male	19.84±0.83^c^	2.64	20.49±0.27^c^	1.15	22.61±0.38^b^	1.21	19.82±0.32^c^	1.00	27.91±0.39^a^	1.25	21.91±0.43	3.27
	Female	17.27±0.28^c^	1.59	17.22±0.11^c^	0.62	19.49±0.15^b^	0.85	17.46±0.12^c^	0.68	24.42±0.24^a^	1.29	19.15±0.24	2.96
Breast length (cm)	Male	11.22±0.14^c^	0.45	11.02±0.15^c^	0.64	12.55±0.18^b^	0.57	10.50±0.26^d^	0.82	13.60±0.15^a^	0.48	11.67±0.16	1.24
	Female	9.46±0.08^c^	0.48	9.15±0.11^c^	0.58	10.69±0.13^b^	0.72	9.31±0.11^c^	0.58	12.02±0.12^a^	0.65	10.12±0.10	1.24
Femur length (cm)	Male	10.05±0.18^c^	0.58	9.34±0.16^d^	0.66	10.86±0.16b	0.52	10.18±0.25^c^	0.79	12.30±0.36^a^	1.15	10.38±0.16	1.26
	Female	9.25±0.12^bc^	0.69	8.34±0.12^d^	0.68	9.46±0.11^b^	0.61	8.92±0.12^c^	0.67	10.64±0.15^a^	0.84	9.32±0.08	1.02
Tibia length (cm)	Male	12.48±0.27^c^	0.86	14.65±0.27^b^	1.15	14.87±0.22^b^	0.70	13.52±0.19^c^	0.60	18.03±0.43^a^	1.37	14.87±0.24	1.84
	Female	10.52±0.10^d^	0.55	12.16±0.11^bc^	0.60	12.37±0.13^b^	0.72	11.82±0.16^c^	0.90	15.21±0.15^a^	0.84	12.39±0.14	1.71
Shank length (cm)	Male	9.59±0.19^b^	0.60	8.89±0.11c	0.48	9.44±0.19^bc^	0.62	9.43±0.33^bc^	1.05	11.19±0.18^a^	0.58	9.60±0.13	1.01
	Female	7.88±0.11^b^	0.64	7.06±0.07^d^	0.39	7.54±0.07^c^	0.38	7.33±0.09^c^	0.50	9.11±0.12^a^	0.65	7.79±0.07	0.88
Shank diameter (cm)	Male	1.31±0.08^bc^	0.25	1.25±0.02^bc^	0.11	1.36±0.04^b^	0.12	1.18±0.05^c^	0.15	1.53±0.04^a^	0.14	1.32±0.02	0.18
	Female	1.01±0.02^c^	0.09	0.97±0.01^c^	0.06	1.08±0.01^b^	0.07	0.92±0.01^d^	0.07	1.26±0.02^a^	0.11	1.05±0.01	0.14
Third finger length (cm)	Male	5.14±0.07^bc^	0.23	4.87±0.09^c^	0.38	5.43±0.09^b^	0.28	5.26±0.11^b^	0.34	6.67±0.15^a^	0.48	5.39±0.09	0.71
	Female	4.54±0.07^c^	0.42	4.13±0.06^d^	0.32	4.74±0.06^b^	0.34	4.60±0.06^bc^	0.30	5.71±0.06^a^	0.36	4.74±0.05	0.62

Superscript mean in the same rows with different superscripts is significantly different (p<0.05)

### PCA

**Figure-4:**
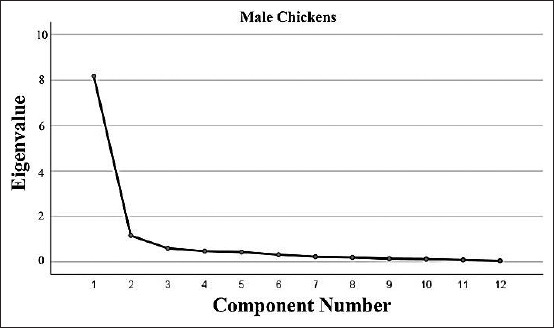
Scree plot showing component number with eigenvalues for quantitative traits of male chickens.

**Figure-5:**
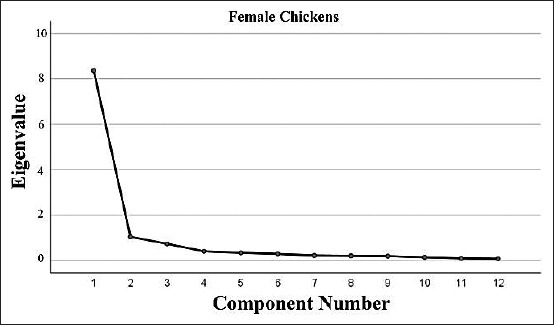
Scree plot showing component number with eigenvalues for quantitative traits of female chickens.

Scree plots of the component number with eigenvalues for the quantitative traits of male and female chickens are presented in Figures-4 and 5. The Eigen values, percentage of the total variance, rotated component matrix, and communalities for all quantitative traits are tabulated in Table-3. In Black Kedu chickens, four components were extracted, which accounted for 84.18% (male) and 72.99% (female) of the total variance in the original variables. PC1 had high positive loadings on body weight (0.915 in males and 0.920 in females). Body measurements, such as breast circumference, wing length, and shank length also had high loadings on PC1 in both sex groups. In Gaga chickens, three (in males) and four (in females) principal components were extracted, which accounted for 68.77% and 67.86%, respectively, of the total variance in the original variables. PC1 had high loadings for body weight (0.920 in male and 0.756 in female) alone. In Merawang chickens, five (males) and four (females) principal components were extracted, which accounted for 91.56% and 67.82%, respectively, of the total variance in the original variables. PC1 was generally correlated with body weight, beak length, and tibia length. In Nunukan chickens, three (male) and four (female) components were extracted, which accounted for 84.35% and 72.75%, respectively, of the total variance present in the seven original variables. PC1 was highly correlated with body weight (0.929 in males and 0.898 in females). Wattle length, breast circumference, and breast length were also correlated in PC1. In Pelung chickens, four (males) and three (females) principal components were extracted, which accounted for 84.87% and 68.77%, respectively, of the total variance present in the seven original variables. Body weight, wattle length, and breast width were correlated in PC1.

**Table-3:** Eigenvalues, percentage of total variance, rotated component matrix, and communalities of quantitative traits observed in Indonesian indigenous chicken breeds.

Breed	Trait	Male						Female				
	
		PC1	PC2	PC3	PC4	PC5	Communalities	PC1	PC2	PC3	PC4	Communalities
Black Kedu	Body weight	0.915	−0.197	−0.065	−0.006	-	0.881	0.920	0.072	0.024	−0.012	0.852
	Beak length	0.421	0.484	0.721	0.073	-	0.937	−0.179	0.697	0.094	−0.533	0.811
	Wattle length	0.704	−0.117	0.008	0.315	-	0.608	0.187	0.272	−0.623	0.609	0.867
	Breast width	0.640	−0.570	−0.232	−0.225	-	0.839	0.778	−0.040	−0.113	−0.255	0.685
	Breast circumference	0.767	−0.320	−0.332	−0.034	-	0.802	0.890	0.026	−0.016	0.152	0.816
	Wings length	0.881	0.019	−0.043	−0.027	-	0.779	0.799	−0.146	0.182	−0.110	0.706
	Breast length	0.368	0.246	−0.113	0.784	0.824	0.559	0.193	−0.603	−0.186	0.747
	Femur length	0.252	−0.663	0.559	0.315	-	0.914	0.617	−0.136	−0.105	−0.402	0.571
	Tibia length	0.607	0.731	0.041	−0.028	-	0.906	−0.072	0.723	0.397	0.251	0.748
	Shank length	0.935	−0.009	−0.089	0.026	-	0.883	0.772	0.368	0.057	0.030	0.734
	Shank diameter	0.469	0.682	−0.308	−0.184	-	0.814	0.548	−0.037	0.277	0.397	0.536
	Third finger length	0.623	−0.044	0.474	−0.549	-	0.916	0.659	−0.155	0.459	0.125	0.684
	Eigenvalues	5.333	2.217	1.345	1.208			4.978	1.327	1.266	1.188	
	% of total variance	44.441	18.472	11.205	10.066			41.484	11.059	10.553	9.898	
Gaga	Body weight	0.920	−0.147	−0.019	-	-	0.869	0.756	0.468	−0.003	0.042	0.793
	Beak length	0.302	−0.496	0.507	-	-	0.594	0.273	−0.557	−0.339	0.477	0.727
	Wattle length	0.711	−0.345	0.174	-	-	0.655	−0.147	0.544	−0.083	0.592	0.675
	Breast width	0.704	−0.307	0.312	−	-	0.687	0.550	0.458	−0.213	−0.220	0.606
	Breast circumference	0.836	−0.038	0.008	-	-	0.700	0.328	0.550	0.058	−0.192	0.450
	Wings length	0.816	0.254	−0.077	-	-	0.736	0.688	0.280	0.271	0.077	0.631
	Breast length	0.683	−0.055	−0.256	-	-	0.535	0.824	0.165	0.087	0.157	0.738
	Femur length	0.738	−0.289	−0.250	-	-	0.690	0.478	−0.469	0.024	−0.283	0.529
	Tibia length	0.535	0.717	0.239	-	-	0.857	0.729	−0.309	−0.440	0.230	0.874
	Shank length	0.542	0.028	−0.603	-	-	0.658	0.725	−0.348	0.185	0.129	0.697
	Shank diameter	0.697	0.183	−0.112	-	-	0.531	0.420	−0.176	−0.320	−0.526	0.586
	Third finger length	0.556	0.533	0.383	-	-	0.739	0.297	−0.363	0.781	0.072	0.835
	Eigenvalues	5.688	1.466	1.098				3.780	2.043	1.192	1.128	
	% of total variance	47.404	12.215	9.149				31.502	17.025	9.930	9.398	
Merawang	Body weight	0.548	0.383	0.461	0.212	−0.399	0.864	0.738	−0.103	0.412	−0.189	0.761
	Beak length	0.708	−0.176	0.564	0.141	0.328	0.978	0.596	−0.125	−0.501	0.001	0.623
	Wattle length	0.328	0.346	0.443	−0.555	−0.315	0.831	0.014	0.596	0.605	0.243	0.780
	Breast width	−0.492	0.496	−0.028	−0.240	0.628	0.941	0.527	−0.0068	0.603	−0.052	0.649
	Breast circumference	0.290	0.290	−0.489	0.625	0.075	0.804	0.475	−0.467	0.356	−0.336	0.683
	Wings length	0.319	−0.872	0.123	0.041	0.242	0.939	0.379	0.344	−0.373	−0.499	0.650
	Breast length	0.047	−0.370	0.556	0.693	0.174	0.959	0.626	0.272	−0.312	−0.136	0.582
	Femur length	0.320	−0.758	−0.168	−0.422	−0.026	0.884	0.629	−0.502	−0.096	0.318	0.758
	Tibia length	0.608	0.208	0.037	−0.479	0.544	0.940	0.765	0.019	0.082	−0.105	0.604
	Shank length	0.864	0.113	−0.291	−0.192	−0.157	0.906	0.374	0.709	0.054	0.227	0.697
	Shank diameter	0.589	0.674	−0.168	0.292	0.203	0.957	0.533	0.446	−0.177	0.029	0.516
	Third finger length	−0.416	0.294	0.845	−0.098	0.035	0.984	0.547	−0.186	−0.166	0.689	0.837
	Eigenvalues	3.078	2.699	2.148	1.843	1.219		3.645	1.783	1.592	1.118	
	% of total variance	25.653	22.488	17.897	15.359	10.161		30.376	14.862	13.265	9.319	
Nunukan	Body weight	0.929	0.155	−0.169	-	-	0.916	0.898	−0.196	0.083	−0.104	0.862
	Beak length	0.634	−0.649	−0.138	-	-	0.843	0.403	0.103	−0.487	−0.468	0.629
	Wattle length	0.798	0.353	−0.367	-	-	0.896	0.803	−0.320	0.266	−0.133	0.836
	Breast width	0.453	0.772	−0.149	-	-	0.823	0.611	−0.074	−0.558	0.372	0.829
	Breast circumference	0.907	−0.006	−0.203	-	-	0.864	0.735	−0.450	0.028	0.146	0.765
	Wings length	0.338	0.584	0.672	-	-	0.906	0.311	−0.121	0.610	0.491	0.725
	Breast length	0.853	0.139	−0.076	-	-	0.752	0.764	0.341	0.097	0.001	0.710
	Femur length	0.938	−0.161	−0.063	-	-	0.909	0.736	0.068	0.002	−0.154	0.570
	Tibia length	0.668	−0.146	0.425	-	-	0.647	0.708	0.488	−0.188	0.047	0.777
	Shank length	0.869	−0.368	0.022	-	-	0.892	0.381	0.522	0.505	−0.412	0.841
	Shank diameter	0.839	0.275	0.132	-	-	0.796	0.436	−0.501	−0.133	−0.058	0.462
	Third finger length	0.715	−0.470	0.380	-	-	0.877	0.297	0.569	−0.130	0.547	0.727
	Eigenvalues	7.067	2.006	1.049				4.674	1.580	1.333	1.146	
	% of total variance	58.892	16.716	8.744				38.946	13.166	11.104	9.553	
Pelung	Body weight	0.749	0.536	0.156	−0.209	-	0.916	0.920	−0.147	−0.019	-	0.869
	Beak length	0.580	0.411	−0.181	0.541	-	0.830	0.302	−0.496	0.507	-	0.594
	Wattle length	0.833	0.314	0.019	−0.297	-	0.881	0.711	−0.345	0.174	-	0.655
	Breast width	0.735	0.233	0.201	0.216	-	0.682	0.704	−0.307	0.312	-	0.687
	Breast circumference	0.449	−0.107	0.783	−0.368	-	0.961	0.836	−0.038	0.008	-	0.700
	Wings length	−0.617	0.579	0.113	0.249	-	0.791	0.816	0.254	−0.077	-	0.736
	Breast length	−0.254	0.785	0.008	−0.391	-	0.833	0.683	−0.055	−0.256	-	0.535
	Femur length	0.296	−0.083	0.753	0.425	-	0.842	0.738	−0.289	−0.250	-	0.690
	Tibia length	−0.533	0.801	−0.043	0.116	-	0.941	0.535	0.717	0.239	-	0.857
	Shank length	−0.649	0.602	0.350	−0.051	-	0.909	0.542	0.028	−0.603	-	0.658
	Shank diameter	0.765	0.289	−0.202	0.297	-	0.797	0.697	0.183	−0.112	-	0.531
	Third finger length	−0.683	−0.225	0.467	0.254	-	0.801	0.556	0.533	0.383	-	0.739
	Eigenvalues	4.624	2.717	1.674	1.170			5.688	1.466	1.098		
	% of total variance	38.531	22.638	13.951	9.752			47.404	12.215	9.149		
Overall	Body weight	0.949	0.187	-	-	-	0.936	0.942	0.175	-	-	0.917
	Beak length	0.720	−0.019	-	-	-	0.519	0.674	−0.266	-	-	0.525
	Wattle length	0.573	0.726	-	-	-	0.856	0.332	0.901	-	-	0.921
	Breast width	0.874	0.062	-	-	-	0.769	0.903	0.127	-	-	0.832
	Breast circumference	0.874	0.239	-	-	-	0.821	0.934	0.058	-	-	0.876
	Wings length	0.907	−0.100	-	-	-	0.832	0.943	−0.079	-	-	0.896
	Breast length	0.920	0.005	-	-	-	0.846	0.916	0.021	-	-	0.840
	Femur length	0.813	−0.360	-	-	-	0.791	0.815	−0.059	-	-	0.668
	Tibia length	0.863	−0.045	-	-	-	0.747	0.839	−0.188	-	-	0.739
	Shank length	0.800	−0.357	-	-	-	0.766	0.814	−0.065	-	-	0.667
	Shank diameter	0.725	0.289	-	-	-	0.609	0.870	0.062	-	-	0.760
	Third finger length	0.811	−0.432	-	-	-	0.844	0.845	−0.228	-	-	0.766
	Eigenvalues	8.172	1.163	-	-	-		8.371	1.038			
	% of total variance	68.104	9.695	-	-	-		69.758	8.625			

PCA was also performed for all breeds. In the overall males, two principal components were extracted, which accounted for 77.80% of the total variance in the original variables, with eigenvalues of 8.172 and 1.163, respectively. PC1 had high positive loadings on all traits measured (≥0.720), except for wattle length (0.573). High loading was only observed for wattle length (0.726) in PC2, whereas negative loadings were observed for beak length (−0.019), wing length (−0.100), femur length (0.360), tibia length (−0.045), shank length (−0.357), and third finger length (−0.432) in PC2. In the overall females, of the total variance, 78.38% was accounted for by two principal components, with eigenvalues of 8.371 (PC1) and 1.038 (PC2). PC1 had high positive loadings on all traits measured (≥0.814), except on beak length (0.674), and wattle length (0.332). Negative loadings were observed in PC2 for some traits, including beak length, wing length, femur length, tibia length, shank length, and third finger length. Wattle length was the only variable to have high positive loading (0.901) in PC2.

## Discussion

### Variation in qualitative traits

#### Plumage color

There was no variation in plumage color observed in Black Kedu, Merawang, and Nunukan chickens for both sexes, of which all the chickens possessed multicolor plumage. Asmara *et al*. [7] found that black was the most predominant plumage color in female Pelung chickens. Meanwhile, other studies have found brown as the common plumage color in Ethiopian chickens [15] and Nigerian chickens [16] and white plumage color as a common feature in Ghanaian chickens [8]. A little variation in plumage color was observed in this study, which indicated intensive selection and breeding programs directed toward this trait.

#### Plumage pattern

Our results are in agreement with a previous finding by Syakir [17], who observed wild as the most frequent plumage pattern in Gaga chickens. A study by Asmara *et al*. [7] found that black was the most predominant plumage pattern in female Pelung chickens, but in our study, black ranked third.

#### Feather flick

There were no variations in feather flick as observed in Merawang and Nunukan chickens, of which all males and females had gold feather flick. All Black Kedu chickens in both sexes and all female Sentul chickens had silver feather flick. Variation in the feather flick color could be affected by chickens being raised at different locations. The feather flick of chickens raised in locations with a high intensity of sunlight could be lighter than those kept in a closed house or under roof pen. A previous study by Sopiyana *et al*. [18] found that ducks kept near the coast with a higher intensity of sunlight appear to gleam more.

#### Comb types

Similar to the results of our study, the highest proportion of single comb was also observed in Kedu chickens by Johari *et al*. [19] and in female Pelung chickens by Asmara *et al*. [7]. A single comb was also common in indigenous chickens in Sri Lanka [20], Bangladesh [21], and Nigeria [22,23]. Combs are important structures for heat loss, and they reduce heat levels through convection [24]. As the tropical climate in Indonesia is predominantly characterized by high ambient temperature, a single comb would be beneficial for heat dissipation through the process of vasodilatation.

#### Shank color

Variations in shank color, such as gray-green, black, white, green, black-white, and black-yellow green as observed in this study are similar to the variants observed in Black Kedu chickens [19] and Sri Lanka chickens [20]. No variations for this trait were found in Merawang and Nunukan chicken breeds for both sexes, of which all the samples had a yellow shank. Faruque *et al*. [21] detected wide variations in shank color in Bangladeshi chickens. They were white, yellow, black, and greenish. Asmara *et al*. [7] observed black shank as being the most frequent in female Pelung chickens, which is in contrast to our findings. Variations in shank color are primarily affected by the nutrition of feed sources containing carotene [9].

#### Comb color

Similar to the present findings, red comb was a common feature of indigenous chickens in Bangladesh [21], Algeria [25], Tanzania [26], and Sri Lanka [20]. As red coloration is an important indicator of the quality of sperm in male birds [27], our findings might indicate good fertility in the chickens investigated.

#### Ear lobe

Most (73.53% in males and 79.12% in females) of the chickens had red earlobes. As noted by some published reports, red and white or their combination are the most common earlobe color in many indigenous chickens, including in Algeria [25], Ethiopia [15], Bangladesh [21], and Sri Lanka [20]. However, in Mediterranean regions, white-earlobes are dominant in chickens [9].

#### Eye color

Orange was the most predominant eye color in this study. Similarly, orange eyes are also a common feature in Algerian chickens [24]. In contrast, Negassa *et al*. [9] observed black-blue as the most predominant eye color in indigenous Ethiopian chickens. Meanwhile, Asmara *et al*. [7] found three variants of eye color in female Pelung chickens: Yellow, red, and black. Eye color in chickens is affected by carotenoids, and melanin in the blood may cause changes in the surface of the iris, which results in different iris colors and may indicate different breeds of chickens. The red color of iris chickens is due to the presence of capillaries from which blood flows, while the yellow color is due to carotenoid deposition [28].

### Variation in quantitative traits

The overall mean values for body weight, breast circumference, wing length, breast length, and tibia length in Merawang chickens were higher than those reported by Hidayat *et al*. [6]. In addition, Sartika *et al*. [29] obtained higher body weight and body measurements for Nunukan chickens than our results. Rachma *et al*. [5] found the lower body weight in Gaga chickens, while Ashifudin *et al*. [30] found the higher body weight and body size in Kedu chicken than we did. Mean body weight and body measurements were also comparable with those reported for indigenous chickens from other countries, though agro-climatic conditions in each region and the age of the sampled chickens may affect the results. Mean body weight and shank length in this study were relatively similar to those reported for local chickens in Ghana [8], but they were relatively higher than those reported for Nigerian [16] and Ethiopian [9] chickens. Compared with the current results, Dahloum *et al*. [25] observed the lower body weight and higher beak length in Algerian chickens, while Faruque *et al*. [21] found a higher shank length in Bangladeshi chickens.

The variations in quantitative traits compared with the previous studies could be associated with the genetic background of the indigenous chickens, as well as the quality and quantity of feed resources available in the different regions where the chickens are reared. However, these variations could provide valuable information for the design of genetic improvement and selection programs for chickens, which depend primarily on the variations present within and among breeds or populations.

### PCA

In this study, the PCA was performed separately for each chicken breed. Data on quantitative traits were subjected to KMO, which was computed to test the sampling adequacy and the pattern of correlation among the traits, and Bathlett’s test was used to assess the validity of the factor analysis of each of the datasets and to test whether the partial correlations among the traits were small. A KMO value of 0.50 and above was considered adequate [14]. In this study, the KMO values were 0.914 and 0.948 for male and female chickens, respectively. These values indicated that the sample size was adequate to apply PCA. The Bartlett’s test of sphericity results for all quantitative traits in male and female chickens was *χ*2=701.46 (p<0.00) and *χ*2=1992.94 (p<0.00), respectively. These values indicated that the datasets were also applicable to perform PCA.

The principal component matrices for all quantitative traits of males and females of each chicken breed are tabulated in Table-3. The coefficients presented indicated the relative contribution of each variable to a particular principal factor, while the percentage of total variance explains how well the total component solutions account for what the variables or measurements represent. Our findings agree with some previous reports, which find that body measurement is generally the primary factor explaining variation. Dahloum *et al*. [25] reported that PC1 includes the general size of the birds, such as body length, tarsus length, and wing span in male chickens, and comb height, breast width, and wing span in female chickens. High positive loadings in PC1 have also been reported for shank length, breast circumference, and body weight in broiler chickens [31]. The observed traits that are loaded in the same component can be classified as the same cluster, which can have common genomic sites for their genetic control. Along with body measurement, body weight has been reported to have high loading in PC1 [30,31]. Notably, in the present study, wattle length had the lowest coefficient for PC1, which is the component that contributes most to the total variance, for either of the extracted components in male (0.573) and female (0.332) chickens, indicating that this trait had a very low contribution to explaining the total variance.

The communalities representing estimates of the variance in each variable observed ranged between 0.519 (beak length) and 0.936 (body weight) in males and between 0.525 (beak length) and 0.921 (wattle length) in females. The relatively high communalities observed in this study are similar to those reported for Algerian chickens (0.57-0.91 in males and 0.55-0.92 in females) [25], Nigerian chickens (0.456-0.963) [32], and broiler chickens (0.413-0.940) [33]. High communalities indicated that the extracted components could explain the variables well.

Our results indicate an important biological aspect underlying the relationships among morphological traits. Therefore, the principal components extracted in this study could provide valuable information for evaluating animals for selection and breeding purposes.

## Conclusion

We found considerable phenotypic variations in the qualitative and quantitative traits of indigenous chicken breeds in Indonesia. Breed had a significant (p<0.05) effect on all quantitative traits measured. Pelung chickens had higher body weight and body measurements than other breeds. However, the phenotypic variations found in this study are unevenly distributed among the breeds, indicating the existence of breed-specific adaptive responses. Therefore, it is recommended that a further molecular characterization should be used to back up the present findings and determine genetic variation within and among the chicken breeds. Finally, both phenotypic and genetic variation should be considered together to develop effective utilization and conservation programs.

## Authors’ Contributions

DM and HS: Designed the study. FM, APZNLS and AF: Collected the samples and performed the study. DM: Drafted the manuscript. DNHH: Analyzed the data. All authors have read and approved the final manuscript

## Supplementary

**Supplementary Table-1:** The details of sampling sites.

Breed	Sampling sites	No. of samples	Geographical position (latitude/longitude)
Black Kedu	Temanggung, Central Java	42	7°17’48.2”S/110°10’47.6”E
Gaga	Bantul, DIY and Kendal, Central Java	48	7°50’50.6”S/110°23’48.7”E and 6°55’30.2”S/110°09’10.8”E
Pelung	Cianjur, West Java	40	6°49’23.5”S/107°07’13.1”E
Sentul	Majalengka, West Java	40	6°43’36.3”S/108°16’41.0”E
Merawang	Bangka islands, Bangka Belitung	40	2°12’30.0”S/105°58’20.7”E
Nunukan	Nunukan, North Kalimantan	40	4°05’48.5”N/117°42’23.9”E
